# Circulating Tumor DNA-Based Molecular Residual Disease in Early-Stage Non-Small-Cell Lung Cancer: Biology, Assays, and the Path Toward MRD-Guided Care

**DOI:** 10.3390/cancers18183043

**Published:** 2026-09-19

**Authors:** Rajan Yadav, Masaoki Ito, Hideki Ujiie, Shuta Ohara, Yasuhiro Tsutani, Goutham Sunny

**Affiliations:** 1 Department of Medical Oncology, GCRI and BJMC, Ahmedabad 380016, India; rajan121@gmail.com (R.Y.); drgouthamsunny@gmail.com (G.S.); 2 Division of Thoracic Surgery, Department of Surgery, Kindai University Faculty of Medicine, Sakai 590-0197, Osaka, Japan; 230400@med.kindai.ac.jp (M.I.); hideki.ujiie.md@gmail.com (H.U.); 154148@med.kindai.ac.jp (S.O.); 3 Department of Thoracic Surgery, Kindai University Nara Hospital, Ikoma 630-0293, Nara, Japan

**Keywords:** circulating tumor DNA, molecular residual disease, non-small-cell lung cancer, liquid biopsy, adjuvant therapy, perioperative immunotherapy, clonal hematopoiesis

## Abstract

Complete surgical resection remains the cornerstone of curative therapy for early-stage non-small-cell lung cancer (NSCLC), yet a substantial proportion of patients relapse despite a macroscopically complete operation and negative conventional staging. This residual risk reflects micrometastatic disease that is below the resolution of computed tomography and that current clinicopathologic risk factors only coarsely predict. Circulating tumor DNA (ctDNA) provides a direct, blood-based readout of this molecular residual disease (MRD), its detection after definitive treatment is among the strongest known predictors of recurrence, and ctDNA frequently rises months before radiographic progression. In this narrative review, based on a literature search closed on 12 September 2026, we synthesize the foundational and translational literature underpinning ctDNA-MRD in resected NSCLC. We outline the biology of ctDNA shedding and metastatic dissemination established by serial-sampling and phylogenetic studies; the prognostic performance of landmark and longitudinal MRD, tabulated quantitatively across the major cohorts, meta-analyses and trial-nested analyses; the perioperative kinetics that dictate when sampling is informative; the analytical design of tumor-informed versus tumor-agnostic assays and the determinants of their sensitivity; and the biological and technical sources of false-negative and false-positive results, of which clonal hematopoiesis and low-shedding histologies are the most consequential. We then position MRD within the contemporary perioperative treatment landscape—adjuvant targeted therapy and immunotherapy, and neoadjuvant and perioperative chemoimmunotherapy—and review the emerging, still-investigational paradigm of MRD-guided escalation and de-escalation, including the early termination of the MRD-selected MERMAID trials. We conclude with the analytical, evidentiary and implementation barriers—and the current positions of guideline bodies and regulators—that separate a powerful prognostic biomarker from a validated predictive tool, and we set out the trial-design requirements needed to close that gap.

## Simple Summary

Surgery can cure early-stage lung cancer, but a substantial number of patients relapse even when the operation removes all visible disease and scans afterwards look normal. The reason is that tiny deposits of cancer, too small for any scan to see, can remain behind. Tumors shed fragments of their DNA into the bloodstream, and very sensitive blood tests can now detect these fragments after surgery. A positive test identifies patients whose cancer is very likely to return, often many months before it becomes visible on imaging, while patients who repeatedly test negative do well. In this review we explain how these tests work, how sensitive they are, why they sometimes give the wrong answer, and how they might be used to decide who needs extra treatment after surgery. We also set out what still needs to be proven before such blood tests can guide everyday care.

## 1. Introduction

Approximately one in five to one in two patients with completely resected NSCLC will ultimately experience recurrence, with risk rising sharply by pathologic stage. For decades the only tools to anticipate this risk were stage, histology, and a small set of molecular and clinical covariates—instruments that stratify populations but perform poorly for the individual patient. The clinical consequence is a pervasive uncertainty at the point of greatest therapeutic leverage: immediately after surgery, when adjuvant therapy can still alter the natural history of disease but is delivered, by necessity, to many patients who are already cured and to some who will relapse regardless.

Circulating tumor DNA offers a way to address this problem directly. Fragments of tumor-derived DNA shed into the bloodstream can, with sufficiently sensitive assays, be detected after curative-intent therapy as a molecular signature of residual disease. The foundational demonstration that ctDNA could be quantified across a broad range of patients using hybrid-capture deep sequencing [1,2] was followed rapidly by proof that this signal carried prognostic meaning in localized lung cancer: post-treatment ctDNA detection identified patients destined to relapse and did so, on average, well before imaging [3]. A decade of subsequent work—culminating in MRD analyses nested within practice-defining trials [4]—has established ctDNA-MRD as arguably the most powerful prognostic biomarker available in resected NSCLC.

This review synthesizes the biological mechanisms of ctDNA shedding, evaluates current assay platforms, and outlines the clinical trial landscape in which MRD must be situated. Throughout, we distinguish what is firmly established—that MRD is profoundly prognostic—from what remains investigational, namely whether acting on MRD improves outcomes, because conflating the two is the principal risk in translating this technology.

This is a narrative rather than a systematic review. We searched PubMed, ClinicalTrials.gov and the proceedings of the ASCO, ESMO and IASLC meetings for English-language reports on ctDNA-based MRD in resected or resectable NSCLC, with a final search on 12 September 2026, and gave priority to prospective cohorts, biomarker analyses nested within phase 3 trials, meta-analyses, head-to-head assay comparisons and consensus statements published in 2024–2026. Several reviews and consensus documents have appeared recently, including the 2026 Delphi consensus of the Asian Thoracic Oncology Research Group (ATORG) [5] and the 2024 final guidance of the U.S. Food and Drug Administration (FDA) on ctDNA in early-stage drug development [6]. The present review differs from these in four respects: (i) it uses the distinction between prognostic and predictive utility as its organizing principle and states, for each proposed application, which of the two has been demonstrated; (ii) it tabulates the major NSCLC MRD studies quantitatively, placing the declared limit of detection (LOD) of each assay alongside sensitivity, specificity, negative predictive value (NPV) and hazard ratios (Table 1); (iii) it incorporates the 2025–2026 evidence, including the ADAURA and AEGEAN MRD analyses, the 5-year CheckMate 816 and IMpower010 results, ultrasensitive whole-genome-informed assays validated in TRACERx, NSCLC-specific meta-analyses, head-to-head assay comparisons and the early termination of the MRD-selected MERMAID trials; and (iv) it closes with specific trial-design requirements rather than a general call for further research.

**Table 1:** Quantitative summary of the principal ctDNA-MRD studies in early-stage NSCLC and of the relevant meta-analyses.

Study [Ref.]	Design; n; Stage	Assay (Declared LOD)	Landmark Sample	Prognostic Performance	Lead Time/NPV
CAPP-seq (Chaudhuri 2017) [3]	Single-center; 40 pts, 255 samples; stage I–III; surgery or RT	Tumor-informed hybrid capture (CAPP-seq)	First post-treatment sample	ctDNA detected in the first post-treatment sample of 94% of patients who later recurred	Median lead 5.2 months (72% preceded imaging)
TRACERx phylogenetic (Abbosh 2017) [7]	Prospective; first 100 pts; stage I–III	Bespoke multiplex PCR (phylogenetic panel)	Serial post-op	ctDNA detected before or at relapse in 13/14 relapsing pts; detectability tracked tumor volume, proliferation, necrosis	Median lead ~70 days
DYNAMIC lung (Chen 2019) [8]	Prospective perioperative kinetics cohort; stage I–III	Tumor-informed deep sequencing	Post-op day 3	ctDNA half-life ~35 min; day-3 ctDNA status predicted RFS	—
LUNGCA-1 (Xia 2022) [9]	Prospective multicenter; 330 pts, 950 samples; stage I–III	Targeted-panel NGS, tumor-informed calling	Day 3 and 1 month	MRD (positive at day 3 and/or 1 month) HR 11.1 for RFS; MRD outranked TNM stage; adjuvant therapy associated with better RFS in MRD-positive (HR 0.3) but not MRD-negative pts (HR 3.1; non-randomized)	—
LUCID/RaDaR (Gale 2022) [10]	Prospective; 88 pts; stage I–III; surgery, RT or CRT	Bespoke tumor-informed (RaDaR, up to 48 variants; ppm-range LOD)	First post-treatment sample	Residual ctDNA after treatment predicted early relapse with high specificity	Detection preceded clinical relapse
Longitudinal undetectable MRD (Zhang 2022) [11]	Prospective; 261 pts; stage I–III	Tumor-informed MRD panel	Landmark plus serial	Longitudinal undetectable MRD defined a “potentially cured” population; detection at any point markedly worse DFS	NPV of longitudinal negativity 96.8%
PROPHET (Chen 2023) [12]	Prospective; 181 pts, 760 samples; stage I–III	Bespoke 50-variant panel; LOD 0.004%	Post-op landmark plus serial	Higher baseline sensitivity (45%) than fixed-panel assays run on the same samples; landmark MRD-positivity prognostic	—
ADAURA MRD (Herbst 2025) [4]	Phase 3 subset; 220 pts; EGFR-mutated IB–IIIA; osimertinib vs. placebo	Tumor-informed personalized assay	Pre-adjuvant baseline plus serial	MRD preceded DFS events; most events occurred after completion of 3 years of osimertinib	Median lead 4.7 months (95% CI 2.2–5.6)
AEGEAN MRD (Reck 2025) [13]	Phase 3 exploratory; ~21% biomarker-evaluable; stage II–IIIB; perioperative durvalumab vs. placebo	Tumor-informed	Adjuvant cycle 1 day 1	12-month DFS 14.3% (MRD-positive) vs. 89.3% (MRD-negative); HR MRD-positive vs. -negative 21.3 (durvalumab) and 14.3 (placebo); no MRD-positive patient had pCR or MPR	Durvalumab vs. placebo: HR 0.56 (MRD-negative), 0.78 (MRD-positive); both CIs cross 1
TRACERx ultrasensitive (Black 2025) [14]	Prospective; 171 pts; stage I–III; pre-operative	WGS-informed (NeXT Personal); LOD 1–3 ppm, 99.9% specificity	Pre-operative	ctDNA detected in 81% of lung adenocarcinomas (53% of stage I); levels < 80 ppm remained prognostic	5-year OS 100% in ctDNA-negative adenocarcinoma vs. 48.8% with high ctDNA
Head-to-head comparison (Cui 2026) [15]	Prospective; 84 pts, 417 samples; stage I–III	Tumor-naïve fixed panel vs. tumor-informed fixed panel vs. personalized panel	Post-op landmark	Landmark sensitivity 13.6%/27.3%/40.9% across the three strategies; personalized panel specificity 100%, PPV 100%; DFS HR 13.98	NPV of personalized landmark test 81.7%
Meta-analysis, resectable cancers (Zheng 2024) [16]	80 prospective studies, 11 tumor types	Mixed	Landmark and longitudinal	Pooled HR for recurrence 7.48 (6.39–8.77); OS HR 5.58	Sensitivity 0.50 (landmark) vs. 0.74 (longitudinal)
Meta-analysis, NSCLC (Lu 2025) [17]	30 studies; 3287 resected pts	Tumor-informed vs. tumor-agnostic	Landmark and longitudinal	Landmark sensitivity 0.42 vs. 0.44; specificity 0.97 vs. 0.93; AUC 0.81 vs. 0.70 (tumor-informed vs. tumor-agnostic)	Differences narrowed with longitudinal testing

AUC, area under the receiver-operating curve; CI, confidence interval; CRT, chemoradiotherapy; HR, hazard ratio; LOD, limit of detection; MPR, major pathologic response; NPV, negative predictive value; pCR, pathologic complete response; PPV, positive predictive value; ppm, parts per million; pts, patients; RFS, recurrence-free survival; RT, radiotherapy; WGS, whole-genome sequencing. Values are those reported by the cited studies; LODs are the analytical values declared by each platform and are not interchangeable across assays.

## 2. Foundations: What MRD Measures and Why It Matters

**Figure 1:**
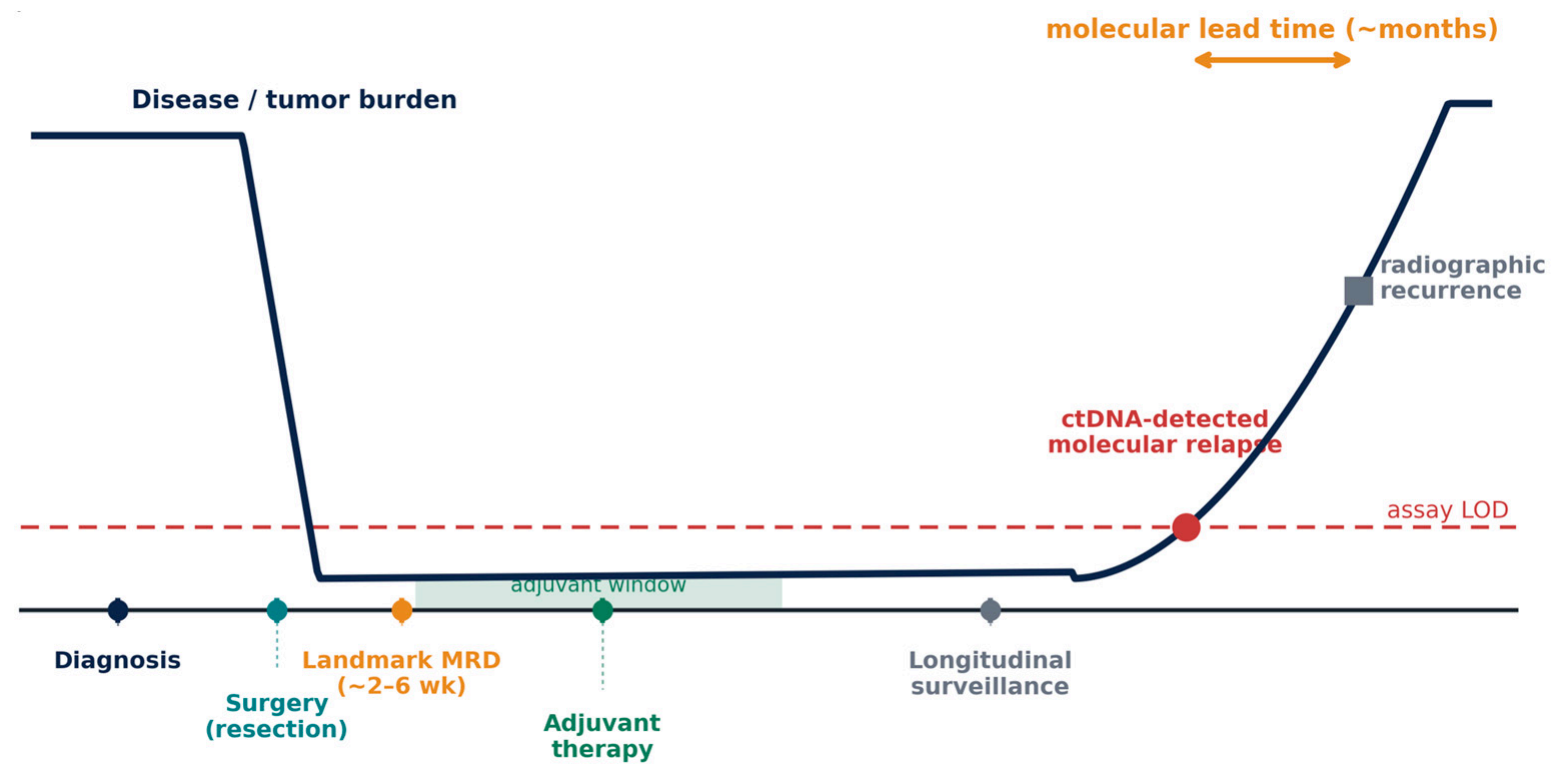
Perioperative ctDNA-MRD timeline and the molecular lead-time window. Schematic of tumor burden across curative-intent treatment for early-stage NSCLC. Burden is high at diagnosis, falls with resection toward an undetectable nadir, and re-expands when micrometastatic disease persists. The dashed line denotes the assay limit of detection (LOD). Sampling points are marked on the time axis: diagnosis, surgery, the landmark MRD window (~2–6 weeks post-operatively), the adjuvant window, and longitudinal surveillance. As burden regrows, ctDNA crosses the LOD and is detected as molecular relapse a median of several months before radiographic recurrence—the molecular lead time. Curve shape, LOD, and lead time are illustrative.

Molecular residual disease denotes tumor-derived material persisting after treatment intended to be curative, below the threshold of conventional imaging. In the ctDNA framework, MRD is operationalized as the detection of tumor-specific somatic alterations in plasma cell-free DNA at or after a defined post-treatment timepoint. Two sampling strategies have emerged (Figure 1). Landmark MRD refers to a single assessment in a narrow post-operative window—typically two to six weeks after resection—chosen to allow surgically released, non-tumor cell-free DNA to clear while preserving lead time. Longitudinal or surveillance MRD refers to serial sampling during follow-up, which captures patients who are MRD-negative at the landmark but later convert as subclinical disease re-expands [11]. Throughout this review, “MRD-positive” means detection above the declared LOD of the assay used; because LODs differ by two to three orders of magnitude between platforms (Section 6), an MRD-negative result is always assay-relative.

The concept of ctDNA-MRD predates its application in lung cancer. Serial mutation tracking predicted relapse in early breast cancer [18] and detected residual disease in resected colorectal cancer [19], establishing the general principle that post-treatment ctDNA is prognostic across solid tumors. The translation to NSCLC introduced a defining challenge that recurs throughout this review: lung tumors, and lung adenocarcinomas in particular, are heterogeneous shedders, such that assay sensitivity and tumor biology jointly determine whether MRD can be detected at all.

## 3. The Biology of ctDNA Shedding and Dissemination

The interpretive foundation of MRD is biological: what does a positive or negative test reflect about the underlying disease? Phylogenetic ctDNA analysis within the TRACERx cohort showed that, in early-stage NSCLC, preoperative ctDNA detection correlates with tumor volume, proliferation, histologic subtype, and the presence of necrosis, and that clonal mutations are shed more reliably than subclonal ones [7]. These findings explain why low-volume, indolent, or lepidic-predominant tumors may be ctDNA-undetectable even when viable disease is present—a biological, not merely technical, limit of detection.

Longitudinal TRACERx analyses subsequently linked post-operative ctDNA dynamics to the biology of metastatic dissemination, demonstrating that ctDNA can track the emergence and clonal composition of relapsing disease and that detection often precedes clinical recurrence by a clinically meaningful interval [20]. Companion analyses of tumor evolution and metastasis in the same cohort provide a phylogenetic framework for interpreting which clones seed relapse [21,22]. Collectively, this body of work reframes ctDNA-MRD not as a binary laboratory result but as a window onto residual tumor burden and its evolutionary trajectory.

## 4. Prognostic Value of MRD in Resected NSCLC

The prognostic signal of ctDNA-MRD in early-stage NSCLC is large and reproducible. The seminal CAPP-seq analysis demonstrated that post-treatment ctDNA detection identified essentially all patients who later relapsed and preceded radiographic progression in the majority [3]. Prospective and multicenter cohorts have since reinforced this: in the LUNGCA-1 study, perioperative ctDNA-based MRD stratified recurrence risk and outperformed conventional clinicopathologic features [9], and the RaDaR analysis showed that residual ctDNA after treatment predicted early relapse with high specificity [10]. Table 1 places the principal NSCLC studies side by side with their design, assay and declared LOD, landmark timing and quantitative performance. Meta-analyses now quantify the effect. Across 80 prospective studies in resectable cancers, postoperative ctDNA positivity carried a pooled hazard ratio (HR) for recurrence of 7.5 (95% CI 6.4–8.8), with a landmark sensitivity of only 0.50 that rose to 0.74 with longitudinal testing [16]; in an NSCLC-specific meta-analysis of 30 studies (3287 patients), landmark sensitivity was 0.42–0.44 for both tumor-informed and tumor-agnostic assays, whereas specificity (0.97 vs. 0.93) and the area under the curve (0.81 vs. 0.70) favored tumor-informed designs [17]. Two features of Table 1 deserve emphasis: the hazard ratios are large and consistent across cohorts, assays and continents, but landmark sensitivities are modest, so that roughly half of the patients who will relapse are MRD-negative at a single post-operative test.

**Figure 2:**
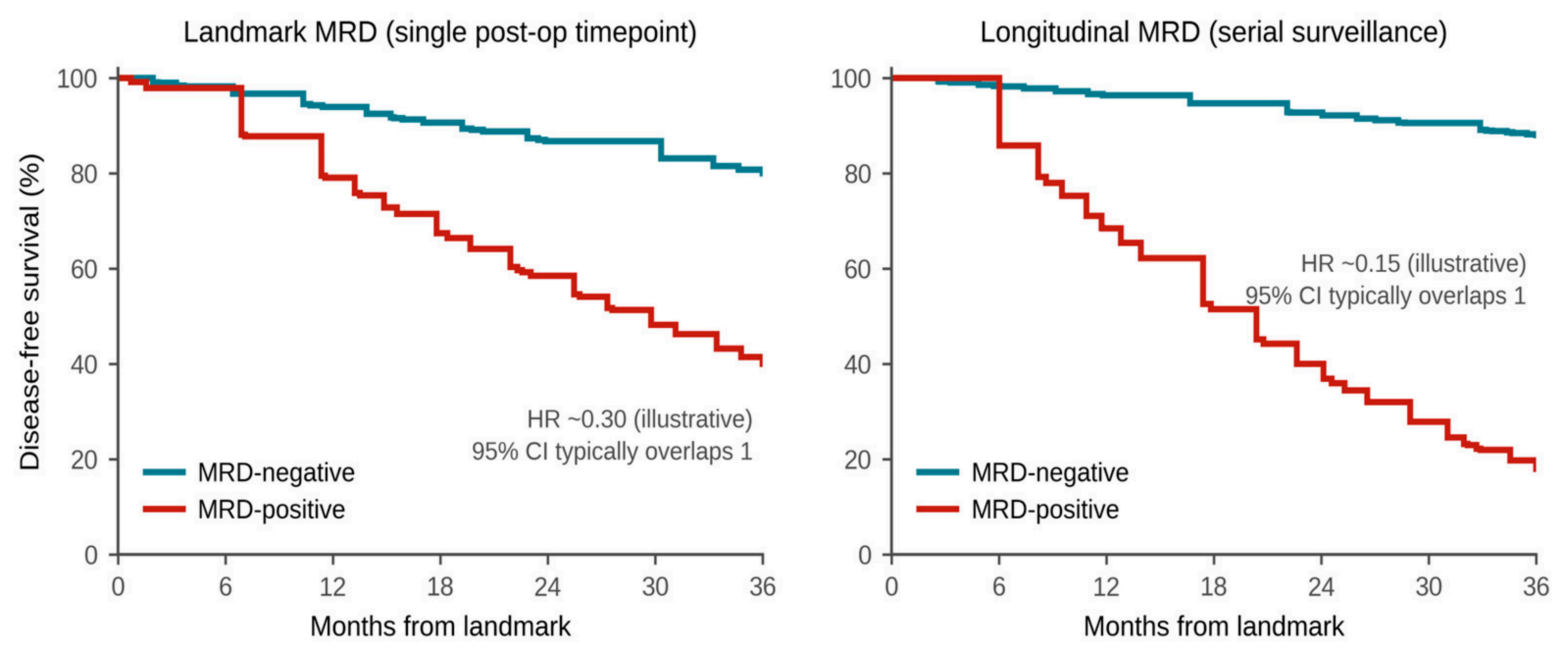
Prognostic stratification by ctDNA-MRD status (schematic Kaplan–Meier). Conceptual disease-free survival curves contrasting MRD-negative and MRD-positive patients for a single post-operative landmark (**left**) and for serial longitudinal surveillance (**right**), which widens separation by capturing later converters. The magnitude of separation and the hazard-ratio annotations are illustrative and deliberately conservative; the curves are schematic and convey the qualitative effect reported across the prognostic literature rather than data from any specific cohort.

A conceptually important refinement is the value of sustained MRD-negativity. Longitudinal analysis demonstrated that patients with durably undetectable MRD across serial timepoints constitute a population with excellent outcomes—operationally, a “potentially cured” group—whereas MRD detection at any point confers markedly inferior disease-free survival [11]. The qualitative effect is summarized in Figure 2: landmark testing already separates risk groups sharply, and longitudinal surveillance widens that separation by reclassifying initially-negative patients who later convert. These data position MRD-negativity as a candidate enrichment criterion for de-escalation and MRD-positivity as a candidate trigger for escalation—hypotheses addressed in Section 9.

## 5. Timing: The Perioperative Kinetics That Govern Detection

When ctDNA is sampled is as consequential as how it is measured (Figure 1). Surgery itself transiently elevates total cell-free DNA through tissue trauma, diluting any tumor signal and depressing the tumor fraction in the immediate post-operative period; sampling too early therefore risks false-negativity, while sampling too late forfeits lead time. Studies of perioperative ctDNA kinetics (the lung DYNAMIC cohort) have characterized this window directly, showing that ctDNA clearance after resection associates with favorable outcomes and that persistence or early reappearance flags high recurrence risk [8]. Earlier feasibility work established that ctDNA detectable preoperatively frequently becomes undetectable after complete resection and that its persistence is ominous [23].

The evidence behind the commonly quoted two-to-six-week landmark window is indirect and deserves to be stated plainly. Three observations support it. First, ctDNA is cleared rapidly: in the lung DYNAMIC cohort the estimated ctDNA half-life after resection was approximately 35 min, so that tumor-derived signal detectable days after surgery reflects ongoing release from residual disease rather than carry-over from the resected tumor [8]. Second, total cell-free DNA rises with surgical tissue injury and falls over the following weeks, so that very early sampling dilutes the tumor fraction; LUNGCA-1, which sampled at both 3 days and 1 month, nevertheless found that positivity at either time point was strongly prognostic (HR 11.1) [9]. Third, the window is what the major trials actually used: 3–4 weeks after surgery in MERMAID-1 [24], the first day of adjuvant therapy in AEGEAN [13] and the pre-adjuvant baseline in ADAURA [4]. The window is therefore a pragmatic convention that balances cell-free DNA clearance against lead time and the need to decide on adjuvant therapy, not a value derived from a formal comparison of time points; the ATORG consensus accordingly calls for harmonization of sampling time points across trials before an optimal landmark can be defined [5].

**Figure 3:**
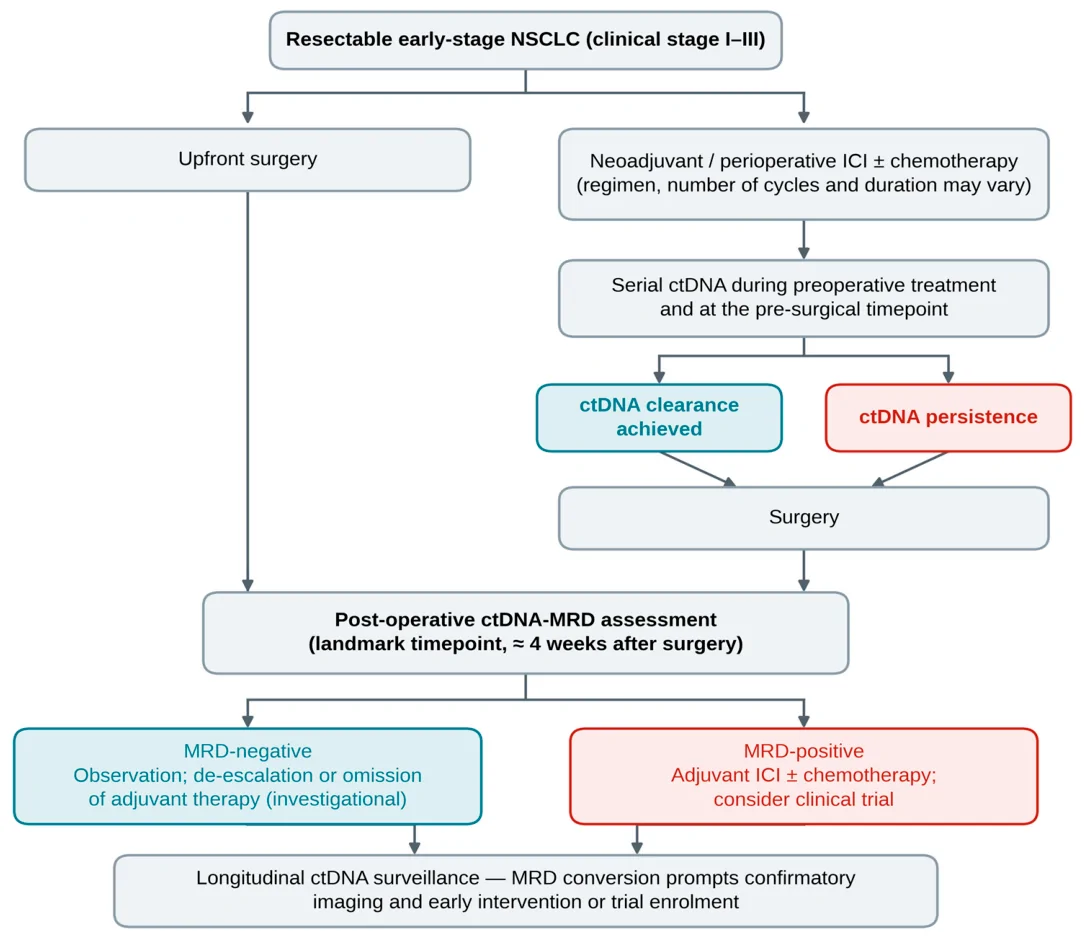
An MRD-guided clinical decision framework for early-stage NSCLC (investigational). Landmark ctDNA-MRD (~2–6 weeks post-operatively) stratifies patients into MRD-positive (escalation/trial enrollment, e.g., MERMAID-type) and MRD-negative (de-escalation/observation under study). All patients enter serial surveillance; MRD conversion flags an opportunity for early intervention before radiographic relapse. The framework organizes hypotheses; MRD-guided escalation/de-escalation in NSCLC is not standard of care.

Quantitatively, the lead time between molecular and radiographic relapse is the parameter of greatest clinical interest because it defines the therapeutic opportunity. In the MRD analysis nested within ADAURA, ctDNA-defined MRD preceded imaging-confirmed disease-free survival events by a median of 4.7 months (95% CI, 2.2–5.6) [4]—consistent in direction with lead times reported across the perioperative literature [3,10,25]. This interval is the biological basis for the decision framework in Figure 3: it is the window in which MRD conversion could, in principle, trigger intervention before macroscopic disease re-emerges.

## 6. Assay Design and Analytical Sensitivity

**Figure 4:**
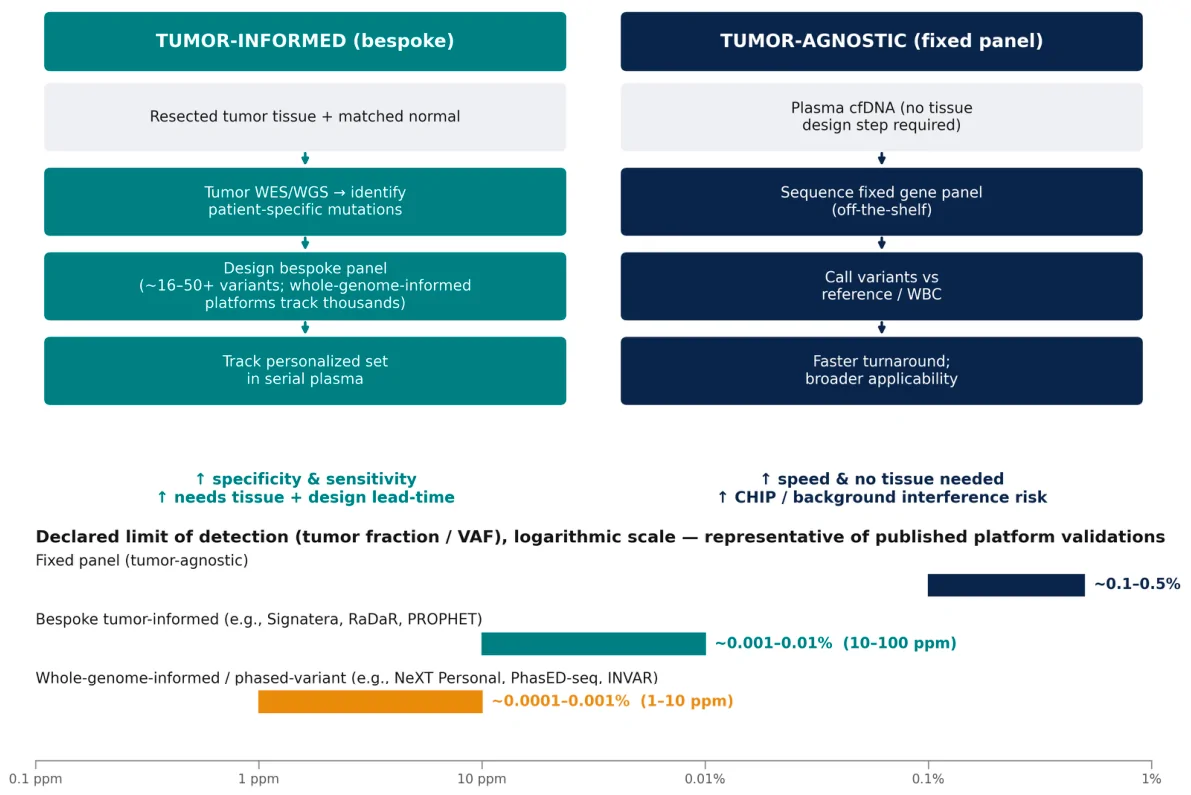
Tumor-informed versus tumor-agnostic ctDNA-MRD assay design. The tumor-informed (bespoke) workflow sequences resected tissue with matched normal to build a patient-specific variant set tracked in serial plasma, maximizing sensitivity and specificity at the cost of tissue and design lead-time. The tumor-agnostic (fixed-panel) workflow interrogates plasma against an off-the-shelf panel, offering speed and broad applicability but greater exposure to background and clonal-hematopoiesis interference. Lower bars give declared limits of detection (tumor fraction/VAF, logarithmic scale) for fixed-panel (~0.1–0.5%), bespoke tumor-informed (~0.001–0.01%) and whole-genome-informed or phased-variant approaches (~1–10 ppm; e.g., NeXT Personal, PhasED-seq, INVAR); values are representative of published platform validations rather than claims for any specific commercial assay.

Two assay philosophies dominate (Figure 4). Tumor-informed (bespoke) assays sequence resected tumor tissue to identify a patient-specific set of somatic variants, then track that fixed set in serial plasma; by concentrating sequencing depth on known true-positive loci, they achieve very low limits of detection and high specificity, at the cost of requiring tissue and a design interval. Tumor-agnostic (fixed-panel) assays interrogate plasma against an off-the-shelf panel without a tissue step, offering faster turnaround and broader applicability but greater exposure to background noise and non-tumor signal. Comparative work in other tumor types has informed the trade-offs between plasma-only and tumor-informed approaches [26]. Table 2 summarizes the principal attributes and trade-offs of the three strategies.

Analytical sensitivity is governed by the interaction of tumor fraction, the number of tracked molecules, and the assay error floor. The methodological progression runs from molecular barcoding and integrated digital error suppression [1,2] through duplex and ultra-rare-mutation sequencing [31,32] to strategies that push detection into the parts-per-million range. Phased-variant detection (PhasED-seq) exploits multiple mutations co-occurring on the same DNA fragment to suppress error and lower the limit of detection by an order of magnitude or more [28]; integration approaches such as INVAR aggregate signal across hundreds to thousands of patient-specific loci [29]; genome-wide and fragmentomic methods—Lung-CLiP for early detection [33], DELFI fragmentation profiling [34], and mutational-integration monitoring [30]—extend sensitivity further; and whole-genome-informed tumor-informed platforms now achieve analytically validated LODs of 1–3 ppm at 99.9% specificity [14]. Three caveats apply to the values in Table 2 and Figure 4. First, declared LODs are analytical, obtained with contrived samples at defined plasma inputs; clinical sensitivity in early-stage NSCLC is far lower because the tumor fraction of many patients lies below any LOD (Section 7). Second, the differences between platforms are real and large: in TRACERx, an assay with an LOD of 1–3 ppm detected pre-operative ctDNA in 81% of lung adenocarcinomas, including 53% of stage I tumors, and ctDNA below 80 ppm—the 95% LOD of the earlier TRACERx assay—remained prognostic for overall survival [14]; in a head-to-head comparison on the same plasma samples, landmark sensitivity rose from 13.6% with a tumor-naïve fixed panel to 27.3% with a tumor-informed fixed panel and 40.9% with a personalized panel, at 100% specificity for the latter [15]. Third, the ranking—fixed panel, then bespoke, then whole-genome-informed or phased—is consistent across the literature, but LODs should always be reported together with the plasma input, the number of variants tracked and the specificity at which they were determined, as the ATORG consensus recommends [5]. Tumor-informed clinical validations in other tumor types (e.g., Signatera [27,35]) provide the analytical template most institutions adopt; the appropriate platform depends on local tissue availability and turnaround requirements.

## 7. Technical and Biological Challenges: False Negatives, False Positives, and Discordance

**Figure 5:**
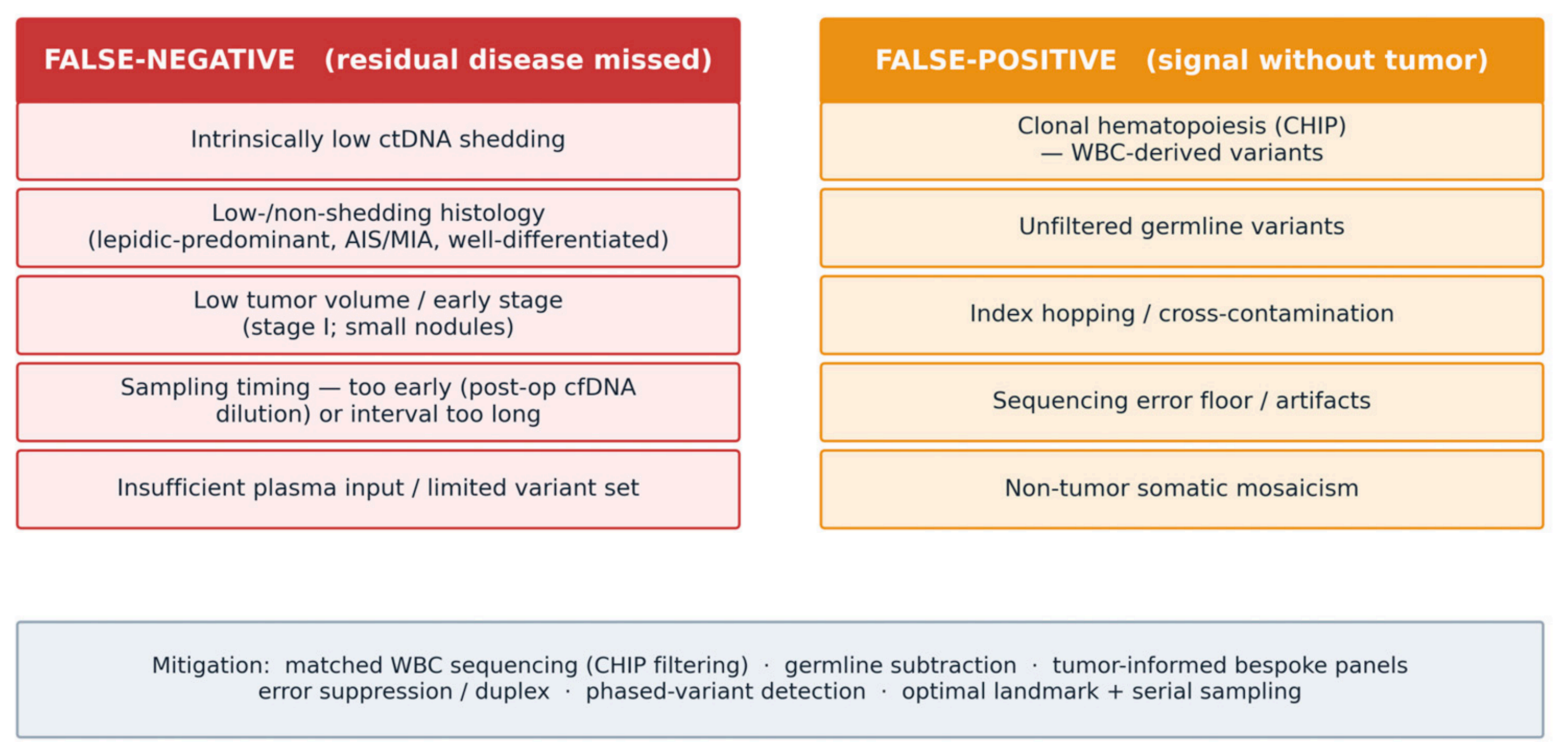
Sources of false-negative and false-positive ctDNA-MRD results. False-negative results (**left**) are predominantly biological: low ctDNA shedding, low-/non-shedding histology (lepidic-predominant, AIS/MIA, well-differentiated), low tumor volume/early stage, suboptimal sampling timing, and limited plasma input or variant sets. False-positive results (**right**) are predominantly technical/non-tumor: clonal hematopoiesis (CHIP), germline variants, contamination/index hopping, sequencing error floor, and non-tumor mosaicism. The lower strip lists mitigations, including matched white-blood-cell sequencing for CHIP filtering.

A clinically actionable MRD result requires understanding the ways it can mislead (Figure 5). False-negativity in NSCLC is predominantly biological. Intrinsically low-shedding tumors, low tumor volume, early stage, and indolent histologies—lepidic-predominant adenocarcinoma and adenocarcinoma in situ or minimally invasive adenocarcinoma—may produce no detectable signal despite viable residual disease, a limitation directly traceable to the shedding biology characterized in TRACERx, in which detectability tracked with tumor volume, proliferation, and histologic subtype [7]. Technical contributors—insufficient plasma input, too few tracked variants, and suboptimal sampling timing—compound these biological constraints.

False-positivity is predominantly technical and non-tumor in origin. The dominant confounder is clonal hematopoiesis of indeterminate potential (CHIP): somatic mutations in hematopoietic cells, which increase with age and are present in a large fraction of older adults [36], release variants into plasma that can masquerade as tumor signal. High-intensity sequencing demonstrated that a substantial share of plasma variants derives from white-blood-cell clones rather than tumor [37], and CHIP has been shown to drive false-positive plasma genotyping in lung cancer specifically [38]. The principal mitigation—matched sequencing of the leukocyte compartment to subtract hematopoietic variants—is now standard in rigorous MRD workflows, alongside germline subtraction, contamination control, error suppression, and bespoke panel design. Discordance between plasma and tissue genotypes, driven by spatial and temporal heterogeneity [39], further argues for tumor-informed designs and cautious interpretation of any single result.

### MRD-Negative Results and Negative Predictive Value

A negative landmark result is the most common result in resected NSCLC and the one most easily misread. Its NPV depends on the sensitivity of the assay, on the prevalence of residual disease in the population tested and on the number of time points sampled. With landmark sensitivities of roughly 40–50% [16,17], a single negative test in a stage II–III population in which 40–50% of patients will relapse yields an NPV of the order of 70–80%—that is, one in four to one in five MRD-negative patients will still relapse. The head-to-head NSCLC comparison reported exactly this: an NPV of 81.7% for the best-performing personalized assay at the landmark [15]. Two strategies raise the NPV. Serial testing captures later converters: in the study of Zhang et al., patients whose MRD remained undetectable across all time points had an NPV of 96.8%, the basis of the “potentially cured” concept [11], and the pan-cancer meta-analysis found a longitudinal sensitivity of 0.74 versus 0.50 for landmark testing [16]. Greater analytical sensitivity does the same: in TRACERx, no patient with lung adenocarcinoma who was ctDNA-negative on an assay with an LOD of 1–3 ppm died within five years [14]. Even at current sensitivities a negative result carries substantial information: in AEGEAN, 12-month DFS was 89.3% in MRD-negative versus 14.3% in MRD-positive patients [13], and in LUNGCA-1 MRD-negative patients derived no apparent benefit from adjuvant therapy [9]. The clinical corollary is that MRD-negativity is a strong but incomplete reassurance; that its use to withhold adjuvant therapy requires either serial negatives or an ultrasensitive assay and, above all, prospective randomized confirmation; and that the NPV should be reported and communicated to patients as a probability rather than as “no residual disease”.

## 8. The Treatment-Defining Trials and Where MRD Fits

The therapeutic context for MRD has been transformed by a succession of positive perioperative trials, which fall into three broad categories; Table 3 lists them together with the ctDNA-MRD analyses each has reported.

### 8.1. Adjuvant Targeted Therapy

ADAURA established three years of adjuvant osimertinib for resected EGFR-mutated stage IB–IIIA NSCLC, with a profound disease-free survival benefit (hazard ratio approximately 0.17 in stage II–IIIA) [40] and a subsequent overall-survival advantage [41]; ALINA extended the targeted-adjuvant paradigm to ALK-positive disease with a similarly large disease-free survival benefit [42]. More recently, the targeted-adjuvant approach has broadened to additional oncogenic drivers: LIBRETTO-432 is evaluating three years of adjuvant selpercatinib in resected stage IB–IIIA RET fusion–positive NSCLC (NCT04819100) [43], and the NCI-led ALCHEMIST program has tested driver-matched adjuvant therapy at scale—erlotinib for EGFR-mutated and crizotinib for ALK-positive disease, alongside the ANVIL nivolumab sub-study—with results to date mixed and several arms still maturing [44]. These settings further widen the post-operative context in which ctDNA-MRD could, in principle, identify the patients most likely to benefit from driver-matched adjuvant therapy.

### 8.2. Adjuvant Immunotherapy

IMpower010 demonstrated a disease-free survival benefit for atezolizumab after chemotherapy, most pronounced in PD-L1-expressing stage II–IIIA tumors [45], a benefit that persisted with at least five years of follow-up [46], and KEYNOTE-091 showed a disease-free survival benefit for adjuvant pembrolizumab in the overall population [47].

### 8.3. Neoadjuvant and Perioperative Chemoimmunotherapy

The neoadjuvant and perioperative settings have moved in parallel. CheckMate 816 established neoadjuvant nivolumab plus chemotherapy, markedly increasing pathologic complete response (approximately 24% vs. 2%) and improving event-free survival [48], and at five years demonstrated an overall-survival benefit (65.4% vs. 55.0%; hazard ratio for death 0.72), with 5-year survival of 95.3% among patients who achieved pathologic complete response [49]. Perioperative regimens that combine neoadjuvant chemoimmunotherapy with adjuvant immunotherapy—KEYNOTE-671 (pembrolizumab) [50] and AEGEAN (durvalumab) [51]—improved event-free survival and pathologic response, and KEYNOTE-671 additionally demonstrated an overall-survival benefit; NADIM II reinforced the strategy in resectable stage III disease [52].

Against this backdrop, MRD offers three complementary roles: as a pharmacodynamic readout of neoadjuvant therapy (ctDNA clearance as an early efficacy signal), as a post-operative risk stratifier to guide adjuvant intensity, and as an enrichment criterion that concentrates trial populations toward patients most likely to benefit. The perioperative trials themselves now supply the most informative NSCLC MRD data, because they combine a randomized comparison with serial sampling. In AEGEAN, only about 21% of patients were biomarker-evaluable with a tumor-informed assay—a limitation shared by every tissue-informed analysis nested in a phase 3 trial—but among them no MRD-positive patient in the durvalumab arm achieved a pathologic complete or major pathologic response, post-surgical MRD-positivity carried a DFS hazard ratio above 14 in both arms, and the effect of durvalumab was directionally similar in MRD-negative (HR 0.56) and MRD-positive (HR 0.78) patients, with confidence intervals crossing unity in both [13]. NADIM II reported the prognostic value of MRD after perioperative chemoimmunotherapy in stage III disease [53], and the ADAURA analysis showed that within the osimertinib arm most disease-free or MRD events occurred after treatment completion and that MRD detection could identify patients who might benefit from longer adjuvant therapy—a hypothesis the authors explicitly flag as requiring prospective confirmation [4]. Together these analyses show that MRD retains its prognostic value in patients treated with immunotherapy and targeted therapy, and that none of them was designed to test, or has shown, that MRD modifies the benefit of treatment.

## 9. From Prognostication to Action: MRD-Guided Escalation and De-Escalation

The central unresolved question is whether acting on MRD improves outcomes. That MRD predicts relapse is established; that intervention triggered by MRD changes the natural history is not. The most instructive precedent comes from colorectal cancer: the randomized DYNAMIC trial (distinct from the lung cohort of the same name cited above) used a ctDNA-guided strategy to direct adjuvant chemotherapy in stage II colon cancer, reducing the proportion of patients treated without compromising recurrence-free survival [55]. DYNAMIC is a proof-of-principle that an MRD-guided strategy can safely de-escalate therapy—but it is cross-tumor, and its result cannot be assumed to transfer to NSCLC, where shedding biology and the efficacy of available systemic therapies differ.

In NSCLC, the escalation hypothesis was taken directly into phase 3, but the test was never completed. The MERMAID program randomized patients selected for MRD-positivity to intensified therapy: MERMAID-1 compared adjuvant durvalumab plus chemotherapy with placebo plus chemotherapy in completely resected stage II–III NSCLC with MRD detected 3–4 weeks after surgery, with DFS in the MRD-positive analysis set as the primary endpoint [24], and MERMAID-2 randomized patients who became MRD-positive during 96 weeks of surveillance after curative-intent therapy to durvalumab or placebo [54]. Both trials were discontinued by the sponsor in 2023, after the approval of perioperative chemoimmunotherapy had changed the standard of care for the target population and made accrual of an immunotherapy-naïve, MRD-positive cohort impractical; MERMAID-1 had randomized 89 of a planned 332 patients and MERMAID-2 34 of a planned 284, and the registries list the studies as completed or prematurely ended with only summary results posted [24,54]. The correct reading is therefore not that MRD-triggered intensification failed, but that it has not been tested: both trials are underpowered for their primary endpoints, and any numerical result from them is inconclusive rather than negative. What the MERMAID experience does establish is a design lesson—MRD-selected trials in NSCLC must be planned around a control arm that will remain standard of care throughout accrual (Section 12). Until adequately powered data mature, MRD-guided escalation or de-escalation in NSCLC remains investigational and should be pursued within trials wherever possible.

The prognostic–predictive distinction can be stated formally. A prognostic biomarker is associated with outcome irrespective of therapy; a predictive biomarker identifies patients in whom a specific treatment changes outcome, which can be demonstrated only by a treatment-by-biomarker interaction in a randomized comparison [6]. Every NSCLC dataset in Table 1 satisfies the first definition; none satisfies the second. The observations most often cited as predictive are exploratory: in LUNGCA-1, adjuvant therapy was associated with better RFS in MRD-positive but not in MRD-negative patients, but treatment was not randomized [9]; in AEGEAN, the point estimates for durvalumab were similar in MRD-negative and MRD-positive patients and both confidence intervals included unity [13]; and in ADAURA, MRD identified patients who might benefit from longer osimertinib, a hypothesis the authors explicitly left for prospective testing [4]. The only randomized demonstration that acting on ctDNA can change management without harm remains the colorectal DYNAMIC trial [55], and it demonstrated safe de-escalation, not a survival benefit from escalation. For the clinician the practical consequence is that an MRD result can inform an estimate of risk and a conversation about surveillance, but cannot by itself justify giving, withholding or prolonging a specific therapy outside a trial [5].

Figure 3 sketches a candidate clinical pathway consistent with current biology: landmark MRD after resection stratifies patients; MRD-positivity motivates escalation or trial enrollment; MRD-negativity may support observation or de-escalation under study; and all patients enter serial surveillance, where MRD conversion flags an opportunity for early intervention within the lead-time window quantified in Section 5. The framework is explicitly aspirational—it organizes hypotheses, not standards of care. Table 4 summarizes what is currently established and what remains investigational.

## 10. Limitations and Unresolved Challenges

Several limitations temper the enthusiasm that ctDNA-MRD has generated (Table 5). The most fundamental is the shedding-dependent sensitivity floor described in Section 3 and Section 7: a negative result cannot be equated with the absence of residual disease, and its negative predictive value is bounded accordingly (Section MRD-Negative Results and Negative Predictive Value). Quantitative claims about sensitivity, the magnitude of prognostic separation, and lead time should be read as direction-of-effect rather than precise estimates, and the schematic figures in this review are deliberately illustrative on this point.

Second, as set out in Section 9, MRD is validated as prognostic but not as predictive, and acting on it outside a trial carries real risks: overtreatment of patients who would never have relapsed, toxicity and cost incurred during the molecular lead-time interval, and the possibility that earlier intervention changes the timing of detection without changing the natural history (lead-time and length-time bias).

Third, the evidence base is uneven in ways that limit generalizability. Much of the most influential data derives from EGFR-enriched or otherwise selected cohorts and from tumor-informed assays validated primarily in other tumor types, and the absence of analytical harmonization across platforms—differing limits of detection, variant-tracking strategies, and reporting conventions—means that results are not freely interchangeable between assays. Standardization of pre-analytical handling, definitions of the landmark window, and reporting thresholds remains incomplete (Section 11).

Finally, this review has its own limitations. It is a narrative rather than a systematic synthesis, and selection of the cited literature, while intended to be representative, is not exhaustive or quantitatively pooled. Several of the trials in Table 3—including LIBRETTO-432 and the ALCHEMIST sub-studies—were ongoing when the literature search closed on 12 September 2026, and their citations reflect that status. The field is moving quickly enough that specific figures, trial readouts, and guideline positions may be superseded between drafting and publication; readers should treat the quantitative specifics accordingly and consult primary sources before acting on them.

## 11. Implementation, Guidelines, and Barriers to Adoption

Guideline bodies have responded with measured endorsement, and their positions can be organized along the evidentiary hierarchy that regulators use. Analytical validity—that an assay measures ctDNA accurately and reproducibly at its declared LOD—is established for the leading tumor-informed platforms but is assay-specific and not transferable between them. Clinical validity—that the result is associated with recurrence—is established for NSCLC by the studies in Table 1 and by the meta-analyses. Clinical utility—that using the result to change treatment improves outcomes—has not been demonstrated in NSCLC. The ESMO Precision Medicine Working Group recommendations [56], the ASCO–CAP joint review [57] and the IASLC statements [58] all stop at the second step, and the 2026 ATORG Delphi consensus, the first NSCLC-specific consensus on MRD testing, reaches the same conclusion: it endorses MRD as a valuable stratification tool and recommends prospective escalation and de-escalation trials, while calling for minimum analytical-performance thresholds, standardized reporting of LOD, variant allele frequency, sensitivity and specificity, cross-platform benchmarking, harmonized pre-analytical handling and sampling time points, and regional guidelines and clinician education before routine use; it also notes that the NCCN guidelines do not currently recommend ctDNA for MRD assessment in early-stage NSCLC [5]. On the regulatory side, the FDA’s final guidance of November 2024 accepts ctDNA in early-stage drug development for patient selection, enrichment and stratification and as a measure of response, but not yet as an endpoint reasonably likely to predict long-term benefit, and it emphasizes assay standardization and harmonization [6]. The consistent message is that ctDNA-MRD is a validated prognostic biomarker but not yet a validated predictive one, and that clinical decisions should not be made solely on MRD outside of trials.

Practical barriers remain substantial and have been discussed above: the tissue and turnaround requirements of tumor-informed assays, which can collide with the landmark window (Section 5); the sensitivity floor and its implications for negative results (Section MRD-Negative Results and Negative Predictive Value); the specificity safeguards of CHIP filtering, germline subtraction and standardized reporting (Section 7); and the absence of cross-platform harmonization (Section 6). To these must be added reimbursement, unequal access that risks widening rather than narrowing disparities in care, and integration into multidisciplinary workflows; the literature on liquid-biopsy implementation enumerates these challenges [59], and the low proportion of biomarker-evaluable patients in trial-nested analyses (about one in five in AEGEAN [13]) shows how far tissue-informed workflows remain from routine feasibility.

## 12. Future Directions

Three priorities will shape the next phase, and each translates into specific design requirements. First, sensitivity: whole-genome-informed, phased-variant and fragmentomic methods now reach single-digit parts per million [14,28,29,30,33,34], and methylation-based approaches are advancing in parallel; this matters most precisely for the early-stage, low-shedding tumors in which current assays underperform. Trials should therefore specify the assay, its declared LOD and the plasma input, collect matched leukocyte DNA for CHIP filtering, and report results by tumor fraction as well as by binary call, following the reporting standards recommended by ATORG [5]. Second, evidence: predictive utility can be established only by randomized, MRD-stratified designs, and the MERMAID experience defines what they must look like. Escalation trials should randomize MRD-positive patients to the current perioperative standard of care with or without the additional intervention, use a control arm that will remain standard throughout accrual, sample at a harmonized landmark (for example, 3–6 weeks after surgery and before adjuvant therapy) with prespecified serial time points, stratify by stage, histology and PD-L1 or driver status, and be powered for DFS with overall survival as a key secondary endpoint; because only a minority of resected patients are MRD-positive at the landmark, screening cohorts several times larger than the randomized population must be planned. De-escalation trials should define the low-risk group by serial negativity or by an ultrasensitive assay, use a non-inferiority design with a prespecified margin, with recurrence-free survival as the primary endpoint and adjuvant-therapy exposure as a co-primary or key secondary endpoint, and include a safety rule that triggers treatment on MRD conversion. Surveillance trials that intervene at MRD conversion should randomize between early intervention and standard imaging-based follow-up so that lead-time bias is controlled, and should be consistent with the FDA guidance on ctDNA as an enrichment and response biomarker [6]. Single-arm and retrospective analyses, however striking, cannot substitute for these designs. Third, integration: MRD will be most powerful when combined with pathologic response, radiomics, and tumor genomics into composite risk models, rather than used as a stand-alone binary classifier.

## 13. Conclusions

Circulating tumor DNA-based MRD has, in little more than a decade, become the most powerful prognostic biomarker in resected NSCLC, capable of identifying impending relapse months before imaging and of defining a potentially cured population among the MRD-negative. Its biology is increasingly well understood, its assays are approaching the sensitivity that early-stage disease demands, and its interpretive pitfalls—chiefly low-shedding histology and clonal hematopoiesis—are now tractable. What remains is the decisive step from prognostication to action: prospective evidence that MRD-guided intervention improves outcomes. The MRD-selected trials designed to provide it in NSCLC were closed early, and the next generation must be built around the current perioperative standard of care. Until such trials report, MRD should inform—not dictate—perioperative decisions, and its greatest near-term value may be as an enrichment and pharmacodynamic tool that makes the next generation of perioperative trials smaller, faster, and more informative.

## Figures and Tables

**Table 2:** Comparison of ctDNA-MRD assay strategies.

Attribute	Tumor-Informed (Bespoke)	Tumor-Agnostic (Fixed Panel)	Whole-Genome-Informed/Phased-Variant
Tumor tissue	Required (tumor + matched normal)	Not required (plasma only)	Often tumor-informed
Turnaround	Slower (bespoke design step)	Faster (off-the-shelf)	Slower (computationally intensive)
Declared LOD (tumor fraction/VAF) *	~0.001–0.01% (10–100 ppm)	~0.1–0.5%	~0.0001–0.001% (1–10 ppm)
Main strength	High sensitivity and specificity	Speed; broad applicability	Lowest limit of detection
Main limitation	Tissue need and design lead-time	Background/CHIP interference	Complexity; maturing validation

* Declared analytical limits of detection reported by representative platforms under their own validation conditions; see Figure 4. Examples: fixed panels, ~0.1–0.5%; bespoke multiplex-PCR or capture panels (Signatera [27], RaDaR [10], PROPHET [12]), ~0.001–0.01%; whole-genome-informed or phased-variant approaches (NeXT Personal [14], PhasED-seq [28], INVAR [29], MRDetect [30]), ~1–10 ppm. LODs depend on plasma input, the number of variants tracked and the specificity threshold chosen, and are not interchangeable between platforms.

**Table 3:** Practice-defining perioperative NSCLC trials and the ctDNA-MRD analyses reported within them.

Trial [Ref.]	Setting; Population	Regimen	Principal Result	ctDNA/MRD Analysis Reported
ADAURA [40,41]	Adjuvant; resected EGFR-mutated IB–IIIA	Osimertinib 3 years vs. placebo	DFS HR ~0.17 (II–IIIA); overall-survival benefit	Yes: MRD preceded DFS events by a median of 4.7 months; most events after treatment completion [4]
ALINA [42]	Adjuvant; resected ALK-positive IB–IIIA	Alectinib vs. chemotherapy	Large DFS benefit	Not reported
LIBRETTO-432 [43]	Adjuvant; RET fusion-positive IB–IIIA	Selpercatinib vs. placebo	Ongoing	Not reported
ALCHEMIST [44]	Adjuvant; EGFR-mutated, ALK-positive and unselected sub-studies	Erlotinib; crizotinib; nivolumab (ANVIL)	Mixed; several arms maturing	Not reported
IMpower010 [45,46]	Adjuvant after chemotherapy; IB–IIIA	Atezolizumab vs. best supportive care	DFS benefit in PD-L1-positive II–IIIA, maintained at ≥5 years	Not a prespecified MRD analysis
KEYNOTE-091 [47]	Adjuvant with or without chemotherapy; IB–IIIA	Pembrolizumab vs. placebo	DFS benefit in the overall population	Not a prespecified MRD analysis
CheckMate 816 [48,49]	Neoadjuvant; IB–IIIA	Nivolumab + chemotherapy × 3 vs. chemotherapy	pCR 24% vs. 2%; EFS benefit; 5-year OS 65.4% vs. 55.0%	Yes: ctDNA clearance during neoadjuvant therapy associated with pCR and EFS [48]
KEYNOTE-671 [50]	Perioperative; II–IIIB	Pembrolizumab + chemotherapy → pembrolizumab	EFS and overall-survival benefit	Not a prespecified MRD analysis
AEGEAN [13,51]	Perioperative; II–IIIB	Durvalumab + chemotherapy → durvalumab	pCR and EFS benefit	Yes: post-surgical MRD in ~21% biomarker-evaluable pts; 12-month DFS 14.3% vs. 89.3%
NADIM II [52,53]	Perioperative; IIIA–B	Nivolumab + chemotherapy → nivolumab	pCR and survival benefit	Yes: MRD prognostic after perioperative chemoimmunotherapy
MERMAID-1 [24]	Adjuvant; MRD-positive II–III (MRD as eligibility criterion)	Durvalumab + chemotherapy vs. placebo + chemotherapy	Enrolment stopped 2023 after 89 of 332 planned pts randomized; underpowered, inconclusive	MRD-selected design
MERMAID-2 [54]	Surveillance; MRD conversion after curative-intent therapy	Durvalumab vs. placebo	Enrolment stopped 2023 after 34 of 284 planned pts randomized; inconclusive	MRD-emergence-triggered design

DFS, disease-free survival; EFS, event-free survival; HR, hazard ratio; OS, overall survival; pCR, pathologic complete response; pts, patients. Candidate ctDNA-MRD integration points across these settings are neoadjuvant clearance as a response readout, landmark testing to guide adjuvant intensity, surveillance for early relapse capture and MRD-positive enrichment for trial eligibility.

**Table 4:** What is established versus investigational in clinical use of ctDNA-MRD.

Domain	Established	Investigational/Uncertain
Prognostic value	MRD is strongly prognostic for recurrence across perioperative settings	Optimal timepoint and positivity threshold not standardized
Predictive value	No randomized treatment-by-MRD interaction demonstrated in NSCLC	Whether acting on MRD improves outcomes remains unproven; the only randomized precedent (DYNAMIC) is in colorectal cancer
Escalation (MRD-positive)	Provides rationale for trial enrollment and enrichment	Benefit of escalation untested: the MRD-selected MERMAID-1/-2 trials closed early and are underpowered
De-escalation (MRD-negative)	Hypothesis-generating	Safety of withholding or limiting adjuvant therapy under study
Surveillance	Detects molecular relapse before imaging (lead time)	Whether earlier intervention alters natural history (lead-time bias)

**Table 5:** Principal limitations of ctDNA-MRD and their clinical implications.

Limitation	Implication
Shedding-dependent sensitivity floor	A negative result cannot exclude residual disease in low-shedding tumors
Modest landmark sensitivity (~40–50%)	NPV of a single test of the order of 70–80%; serial or ultrasensitive testing needed before de-escalation
Prognostic, not yet predictive	Identifying high risk does not prove that intervention helps
MRD-selected trials closed early (MERMAID-1/-2)	Escalation in MRD-positive patients remains untested; MRD-selected designs must anticipate changes in standard of care
Lead-time/overtreatment risk	Earlier action may not change natural history; risk of overtreating non-relapsers
Selected cohorts; no harmonization	Limited generalizability; assays are not freely interchangeable
Cost, reimbursement, access	Risk of widening rather than narrowing disparities
This review (narrative; illustrative figures)	Quantitative specifics are directional and may be superseded

## Data Availability

No new data were created or analyzed in this study. Data sharing is not applicable to this article.

## Abbreviations

The following abbreviations are used in this manuscript:

AIS	adenocarcinoma in situ
ALK	anaplastic lymphoma kinase
ATORG	Asian Thoracic Oncology Research Group
cfDNA	cell-free DNA
CHIP	clonal hematopoiesis of indeterminate potential
CI	confidence interval
CT	computed tomography
ctDNA	circulating tumor DNA
DFS	disease-free survival
EFS	event-free survival
EGFR	epidermal growth factor receptor
FDA	U.S. Food and Drug Administration
HR	hazard ratio
LOD	limit of detection
MIA	minimally invasive adenocarcinoma
MRD	molecular residual disease
NPV	negative predictive value
NSCLC	non-small-cell lung cancer
OS	overall survival
pCR	pathologic complete response
PD-L1	programmed death-ligand 1
ppm	parts per million
RET	rearranged during transfection
RFS	recurrence-free survival
VAF	variant allele frequency
WGS	whole-genome sequencing
